# Cancer Risk in Collagenous Colitis

**DOI:** 10.3390/jcm8111942

**Published:** 2019-11-11

**Authors:** Johanna K. Larsson, Konstantinos J. Dabos, Peter Höglund, Johan Bohr, Andreas Münch, Andry Giannakou, Artur Nemeth, Gabriele Wurm-Johansson, Ervin Toth, John N. Plevris, Paul Fineron, Anastasios Koulaouzidis, Klas Sjöberg

**Affiliations:** 1 Department of Gastroenterology, Skåne University Hospital, 205 02 Malmö, Sweden; johanna.larsson@med.lu.se; 2 Centre for Liver & Digestive Disorders, the Royal Infirmary of Edinburgh, Edinburgh EH16 4SA, Scotland, UK; kostasophia@yahoo.com (K.J.D.); j.plevris@ed.ac.uk (J.N.P.); akoulaouzidis@hotmail.com (A.K.); 3 Department of Laboratory Medicine, Division of Clinical Chemistry and Pharmacology, SUS, Lund University, 221 85 Lund, Sweden; peter.hoglund@med.lu.se; 4 Department of Medicine, Division of Gastroenterology, Örebro University Hospital, 702 81 Örebro, Sweden; School of Health and Medical Sciences, Örebro University, 701 85 Örebro, Sweden; johan.bohr@regionorebrolan.se; 5 Division of Gastroenterology and Hepatology, Department of Clinical and Experimental Medicine, Faculty of Health Science, Linköpings University, 581 83 Linköping, Sweden; Andreas.Munch@regionostergotland.se; 6 Open University of Cyprus, Faculty of Economics and Management, 1678 Nicosia, Cyprus; andry.gianna@gmail.com; 7 Department of Medicine, Endoscopy Unit, Skåne University Hospital, 205 02 Malmö, Sweden; artur.nemeth@med.lu.se (A.N.); gabriele-wurm@web.de (G.W.-J.); ervin.toth@med.lu.se (E.T.); 8 Pathology Department, Western General Hospital, Edinburgh EH4 2XU , Scotland, UK; paul.fineron@nhtlothian.scot.nhs.uk

**Keywords:** colon cancer, cancer risk, collagenous colitis, lung cancer, microscopic colitis, skin cancer, squamous cell carcinoma

## Abstract

Data on malignancy in patients with collagenous colitis (CC) is scarce. We aimed to determine the incidence of cancers in patients with CC. In a two-stages, observational study, data on cancers in patients diagnosed with CC during 2000–2015, were collected from two cohorts. The risk was calculated according to the age-standardized rate for the first cohort and according to the standardized incidence ratio for the second cohort. The first cohort comprised 738 patients (394 from Scotland and 344 from Sweden; mean age 71 ± 11 and 66 ± 13 years, respectively). The incidence rates for lung cancer (RR 3.9, *p* = 0.001), bladder cancer (RR 9.2, *p* = 0.019), and non-melanoma skin cancer (NMSC) (RR 15, *p* = 0.001) were increased. As the majority of NMSC cases (15/16) came from Sweden, a second Swedish cohort, comprising 1141 patients (863 women, mean age 65 years, range 20–95 years) was collected. There were 93 cancer cases (besides NMSC). The risk for colon cancer was decreased (SIR 0.23, *p*= 0.0087). The risk for cutaneous squamous cell carcinoma was instead markedly increased (SIR 3.27, *p* = 0.001).

## 1. Introduction

Microscopic colitis (MC) is an inflammatory disorder of the colon that causes chronic, watery and non-bloody diarrhoea, occasionally associated with abdominal pain and weight loss. With a predilection for those ≥60 years of age and for females, MC has an incidence rate of approximately 10/100.000 per year [1,2,3]. Macroscopic findings are rare and the diagnosis is confirmed through histopathology [4,5,6]. MC comprises two main histologic subtypes; collagenous colitis (CC) and lymphocytic colitis (LC). Histopathological features of CC include a continuous, thickened sub-epithelial fibrous band (>10 µm) and associated chronic mucosal inflammation. The collagen band contains entrapped capillaries, red blood cells, as well as inflammatory cells. Moreover, damaged epithelial cells appear flattened, mucin-depleted, and irregularly-oriented. Focally, small strips of surface epithelium may lift-off the basement membrane [7]. Patients with CC are considered to have a more symptomatic and long-lasting disease course than those with LC [8].

Chronic inflammation is considered a risk factor for cancer development. Moreover, chronic inflammation may result in cancer development in sites other than the affected organ/system. For instance, the risk of lymphoma in rheumatoid arthritis (RA) is increased by 60% [9]. In patients with inflammatory bowel disease (IBD), there is an increased risk of colorectal cancer (CRC), at least in some subgroups, as well as extra-intestinal cancers such as haematological, bladder, lung as well as skin cancers [10,11,12]. Patients with coeliac disease have a reported increased risk of non-Hodgkin lymphoma, small-bowel cancer, CRC and basal cell carcinoma (BCC) [13]. *Helicobacter pylori* itself contributes to many neoplasias, but studies have shown that the inflammatory response per se contributes to the carcinogenesis as well [14].

Data on the incidence of metachronous cancer(s) in MC is scarce. Although the inflammation is limited as compared to classical IBD the condition may be active for several years; furthermore, it often affects elderly individuals who have already an increased cancer risk. Additionally, many patients with CC smoke [15]. Chan et al described an increased risk of lung cancer in a small cohort of patients with CC with a mean follow-up time of 7 years. The study included 117 patients, and no cases of CRC were described [16]. A negative association has actually been suggested between CRC and MC (including both CC and lymphocytic colitis) in a cohort comprising 647 patients with MC and a mean follow-up time of five years. Twelve MC patients had CRC compared to 27 in a control group of similar size (*p* = 0.015) [17]. Therefore, the aim of the present study was to determine the incidence of metachronous cancer in patients with CC.

## 2. Patients and Methods

**Figure 1:**
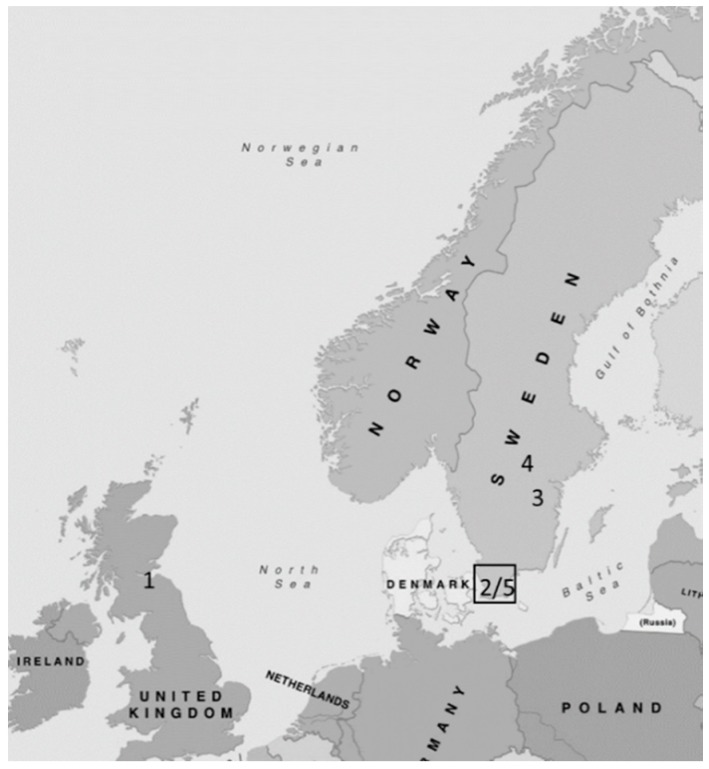
Participating centres: Series 1: 1 = Edinburgh, Scotland, 2 = Malmö; Series 2: 3 = Linköping, 4 = Örebro, 5 = Skåne region.

The investigation was carried out as a two-stage, observational, international, multicentre, cohort-study, comprising two sizeable cohorts of patients diagnosed with CC. See Figure 1.

### 2.1. First Stage—Scotland and Sweden

In an international, retrospective, two-centre observational study; data on extra-colonic cancer in patients with CC were collected for a 14-year period (2000–2013) from Edinburgh, Scotland and Malmö, Sweden. The CC diagnosis was set according to established criteria i.e., symptoms of chronic, non-bloody diarrhoea and histopathological findings of thickened sub-epithelial collagen layer ≥10 µm, associated with chronic inflammation in the lamina propria and with an increased number of intraepithelial lymphocytes [18]. Data were obtained from the pathology department, Edinburgh and Malmö with a catchment area of 750,000 and 320,000 inhabitants, respectively. The records of those with CC were manually searched for data on metachronous, extra-colonic cancers.

### 2.2. Second Stage—Sweden

Due to an unexpectedly skewed distribution of cancer cases in the first stage of the study we decided to re-do the study and focus on Swedish data. Patients with CC in three different regions (Skåne, Linköping and Örebro) were included. In Sweden, all patients that are diagnosed with CC according to the established criteria are registered at the Departments of Pathology and are given a specific code number. All specimens taken during colonoscopy in the three regions are regularly sent to specific Pathology Departments. All patients with a CC diagnosis from 2000 in Skåne and Örebro and from 2008 in Linköping until the end of 2015 were included.

For each patient with CC, the follow-up period began at the time of CC diagnosis and continued until whichever of the following occurred first: death or the end of the observation period (31st December, 2015). Patients were not excluded after their first diagnosis of cancer, since we wanted to examine incidence risk for all cancers developed during follow up. The CC cohort was linked up with the National Cancer Register in each region. Cancers preceding the diagnosis of CC were not included in the cancer data.

### 2.3. Statistical Analysis

#### 2.3.1. First Stage

Person-years at risk was calculated according to age-specific categories up to 85 years. The standard error (Se) was calculated using the Poisson approximation. Confidence interval (CI) of the age-standardised rate (ASR) was compared to public data, available from UK´s National Cancer Intelligence Network. The relative risk (RR) for ASR was calculated and compared to ASR in Lothian region, Scotland. The standardised cancer incidence rates (IR) were also compared to the ones of Lothian under the assumption that populations at the same latitude share the same IR.

#### 2.3.2. Second Stage

Person-years at risk were calculated by gender and 5-year age groups, separately for the 3 geographical areas (Skåne, Linköping, and Örebro). Standardized incidence ratios (SIR) were calculated for each of the reported cancers. Because patients in this cohort came from three different Swedish regions and in light of the known national variations of cancer incidence, the expected numbers of cancers were calculated by pooling the patients and linking each area to existing cancer registries. The expected numbers of cases of cancers and specific cancer types were calculated by multiplying the number of person-years for each gender, age and area group by the corresponding specific cancer incidence rates in the respective areas. SIR and their 95% CI were calculated assuming that the observed number of cases followed a Poisson distribution. Mid-P exact test was applied and values below 0.05 were considered significant. For non-melanoma skin cancer (NMSC), both BCC and cutaneous squamous cell carcinoma (cuSCC) that can occur several times in one individual, the number of tumours was recorded—instead of individual cases—in both the CC cohort and in the control group.

This study was approved by the institutional review board at Lund University (The local Ethics Committee at Lund University, date: 6th November 2013; decision LU 2013/650 and date: 27th October, 2016; decision LU 2016/788) and Lothian NHS. Since it was a retrospective registry study the Ethical Board approved that written informed consent was not necessary to obtain prior to the collection of data. The study protocol conforms to the ethical guidelines of the 1975 Declaration of Helsinki as reflected in a priori approval by the institution’s human research committee.

## 3. Results

### 3.1. First Stage

The demographics of the first cohort can be seen in Table 1. Of the 738 included, 71 (50 women and 21 men) were affected by some form of extra-colonic, metachronous malignancy following the diagnosis of CC. The remainder of this cohort (n = 667) did not develop any extra-colonic cancer during the follow-up period. The average follow-up duration was 7 years (range 2–15 years), while the average time interval between CC and cancer diagnosis was 3 years (range 0–11 years). Of these 71 cases, 14 developed any cancer during the first year, 25 during the following two years and 32 from the third year.

The RR for all cancers, lung, bladder cancer and NMSC, as well as the ASR in patients with CC were higher compared to those of the general population. The RR for lung cancer was 3.88 (CI: 1.62–9.31), for bladder cancer 9.23 (CI: 1.14–75.03) and for NMSC 14.96 (CI: 2.57–87.08). See Table 2.

The cases with bladder and lung cancer were evenly distributed, but in contrast 15/16 cases with NMSC were addressed from the Malmö cohort. Because of this a decision was taken to proceed to a second stage by including more regions in Sweden. Furthermore, in most countries the number of NMSC is difficult to determine because BCC and cuSCC cases are not reported to national cancer registries, or they are reported as one heterogeneous group [19]. However, in Sweden these cancer types are reported separately and consequently it is possible to obtain data on the occurrence of BCC and cuSCC.

### 3.2. Second Stage

In this stage, a total of 1141 patients with diagnosis of CC were identified. It should be noted that 344 out of those from Skåne were included also in stage one. The characteristics of the patients are shown in Table 3. The average follow-up duration was 8 years (range 2–15 years), while the average time interval between CC and cancer diagnosis was 4 years (range 0–14 years). Of the 93 solid cancers 17 were diagnosed during the first year, 25 during the following two years and 51 thereafter. The expected and observed cancer cases are presented in Table 4 and Table 5. The risk of lung cancer was increased in Skåne (SIR 1.85 CI: 1.053–3.029, *p* = 0.034) but since there were no other cases of lung cancer in the other two Swedish regions, this did not become significant in the whole group. The total number of cancer cases besides NMSC was 98. However, five rare cancer cases were considered not applicable for data calculation and thus excluded, leaving 93 cancer cases (61 women and 32 men). The total number of NMSC was 140. The mean time interval between CC diagnosis and cuSCC was 5.4 years, range 0.6–12.1 years. Of the 46 cases of cuSCC, four developed cuSCC during the first year, eight during the following two years, and 34 after three years or more.

## 4. Discussion

This is the largest study published to date on the risk of metachronous malignancies in patients diagnosed with CC. In the first stage, it was noted that the risk for lung and bladder cancer was increased in patients with CC diagnosis. Furthermore, the risk of NMSC was also increased in this cohort. In the second stage of this observational, multicentre study comprising a large Swedish cohort we could confirm the decreased risk of colon cancer in patients with CC, as reported in previous studies [16,17]. However, previous studies either included prevalent cases of CRC (15) or comprised a fairly limited number of CC-patients (14). Analysis of the current large cohort from three counties in Sweden indicated that the risk of getting colon cancer was reduced at least four times (from 8.8 expected cases to two observed). Since we do not have information about previous colonoscopies in this elderly population with gastrointestinal complaints it cannot be excluded that this reduced risk hypothetically could be due to pre-emptive polypectomies preceding the diagnostic endoscopy. However, in patients with longstanding albeit low-grade, inflammatory response in the colon, one would instead expect to observe an increased risk of colon cancer. Nevertheless, not only the inflammation is modest, but it may also be protective. For instance, frequent watery diarrhoea reduces the transit time and likely any potential impact from toxic agents. Yen et al. suggest that elevated intraepithelial lymphocytes in the colonic mucosa in patients with MC may have a protective function against carcinogenesis through recruiting delta-gamma T-cells that kill cells undergoing DNA-damage or cell stress [17].

Data from the second series revealed a more than three-fold increase in cuSCC in patients with CC. Except UV-light exposure and immunosuppressive treatment related to organ transplantation, little is known about risk factors contributing to cuSCC [19,20,21]. Evidence that glucocorticoids enhance the risk of cuSCC is limited, but has been described previously [22,23]. The incidence of NMSC (both cuSCC and BCC) is increased in patients with IBD, but likely related to the exposure of immunosuppressive treatment (thiopurines and biologics) [24]. However, Singh et al. also described an increased risk of BCC in men with Crohn’s disease not treated with immunosuppression [25]. Therefore, not only immunosuppressive treatment is related to an elevated risk of NMSC but also the inflammation per se, probably as a result of dysregulation of the immune system. In CC, Günaltay et al have described a decreased production of IL-37 in such patients, indicating a disturbed immune response [26,27]. Thus, the elevated risk of cuSCC in CC may be related to a malfunctioning immune system caused by the disease itself, medication or other not yet known procarcinogenic factors.

The incidence rate of lung cancer was increased in the first stage cohort but not in the second. However, the incidence was increased also in the second series in Skåne but not for the whole Swedish cohort. In Skåne 878 out of the 1141 cases with CC were identified and 14 cases with lung cancer was found (7.57 expected). In contrast to this large cohort the expected incidence of lung cancer in the cities Örebro and Linköping (based on 150,000 inhabitants in each location) were one case in each location. Consequently, the observed incidence with no cases in these two minor cities must be interpreted with caution. The incidence rate in Skåne with around 1.3 million inhabitants is of course more reliable. The risk of urinary bladder and/or ureteric cancer was increased in the first cohort and showed a positive trend in the second, something that strengthens this observation. The association between CC and smoking habits is already well described [15]. Smoking is related to cancer in both lung and bladder which is probably the reason why these incidence rates are elevated in our CC cohort [28,29].

The risk of cancer in oesophagus also showed a positive trend in the second series. Risk factors of oesophagus cancer are among others, smoking, alcohol, hot liquids and HPV-infection [30]. As can be seen, some of the risk factors are shared with CC such as smoking and alcohol [31]. Furthermore, oesophageal cancer is often of squamous cell origin just as in the skin. In this study we did not get detailed information if the oesophagus cancer cases were of squamous cell or adenomatous origin. However, in view of the low number of cases with oesophageal or bladder cancer, despite a fairly large number of CC patients, a definitive conclusion cannot be drawn regarding putative relationship between CC and cancer in oesophagus or bladder.

Some strengths and limitations should be noted; this is the largest study to date concerning the risk of metachronous cancer in patients with a previous diagnosis of CC. In the second cohort, 1141 patients could be included. The studied regions are well defined, and patients are referred to specific hospitals within these regions. All common cancers types in the western world were represented in the present study. In other words, no common cancer type was totally absent. The control group in the Swedish cohort consisted of all cancer cases in the same regions as our cohorts, adjusted for year of onset, gender and age group. This procedure is necessary in order to obtain more reliable results. Furthermore, the cancer registry at the National Board of Health and Welfare covers more than 96% of the cancer cases in Sweden [32]. Since we wanted to investigate if CC can be considered as a risk factor for cancer, we included only incident cases of cancer diagnosed after the CC diagnosis. The outcome of cancer was assessed from day one after the CC-diagnosis. This may cause a risk of including prevalent cancers, but since we know that the time lapse between disease onset and diagnosis of CC may be long, we believe that these factors level out. A majority of the cancers was diagnosed after more than three years making detection bias less probable.

This study also has some limitations that merit consideration. First, nationwide registers do not contain information about lifestyle habits like smoking, alcohol consumption, family history, exercise or other possible confounding factors. We neither had detailed data of disease severity nor medication in our study population. Information about sun exposure and consequently also about the location of the skin tumours would have been valuable. Furthermore, the associations found are not necessarily causal; one disease could lead to another or a not yet known factor besides the studied could lead to both CC and cancer. The retrospective design also limits the conclusions that can be drawn, although a study with a prospective design would have been difficult to finalize.

Consequently, this study could confirm the previously described negative association between CC and colon cancer, an observation of unknown cause. Even though significance was not achieved for cancers in lung, bladder and oesophagus there was a trend indicating that there could be a correlation anyway. A new, so far, unknown association between CC and cuSCC has also been revealed. There are reports about an increase incidence of BCC in coeliac disease and of NMSC in IBD. Consequently, we have to be extra cautious when examining patients with gastrointestinal inflammation in order to reveal any incident skin cancers.

## Figures and Tables

**Table 1:** Characteristics of the collagenous colitis (CC)-cohort in Scotland and Sweden (Series 1).

	Edinburgh	Malmö	Total
N	394	344	738
Age, median (IQR)	68 (57–76)	69 (59–77)	68 (58–77)
% women (n)	68% (268)	83% (285)	75% (553)
IQR = interquartile range			

**Table 2:** Observed and expected cancers in the Scottish/Swedish cohort (series 1). RR, relative risk.

Cancer Type	Cases	Exp	RR	*P*-Value
Skin (NMSC)	16	1	15.0	0.001
Bladder	6	1	9.2	0.019
Lung	18	2	3.9	0.001

NMSC = non-melanoma skin cancer.

**Table 3:** Characteristics of the CC-cohort Sweden, three regions (Series 2).

	Linköping	Örebro	Skåne	Total
N	130	133	878	1141
Age, median (IQR)	66 (57–75)	64 (53–74)	68 (58–76)	67 (57–76)
% women (n)	75% (98)	84% (112)	74% (653)	76% (863)
IQR = interquartile range				

**Table 4:** Observed and expected cancers in the Swedish cohort, divided into Skåne, Linköping and Örebro (Series 2).

Cancer site	Skåne			Linköping			Örebro		
	obs	exp	SIR	obs	exp	SIR	obs	exp	SIR
Eye	1	0.13	7.64	0	0.023	0.00	0	0.011	0.00
Oesophagus	3	0.65	4.60	0	0.066	0.00	0	0.068	0.00
Cervix	2	0.46	4.33	0	0.07	0.00	0	0.079	0.00
CuSCC	36	11.58	3.11	6	1.44	4.18	4	1.060	3.78
Vulva	1	0.29	3.46	0	0.033	0.00	0	0.049	0.00
CNS	2	1.29	1.55	2	0.19	10.30	0	0.17	0.00
r	3	1.3	2.31	1	0.19	5.14	0	0.20	0.00
Stomach	1	1.27	0.79	1	0.14	7.14	1	0.14	7.27
Bladder/Ureter	8	4.29	1.87	0	0.5	0.00	2	0.38	5.30
Rectal/Anus	1	3.20	0.31	4	0.39	10.19	2	0.44	4.55
Pancreas	3	1.50	2.00	0	0.25	0.00	0	0.21	0.00
Lung	14	7.57	1.85	0	0.89	0.00	0	0.84	0.00
Prostate	8	7.56	1.06	1	1.56	0.64	1	0.56	1.78
Leukemia/Myeloma	3	2.93	1.02	0	0.37	0.00	1	0.40	2.52
Melanoma	3	3.92	0.76	2	0.63	3.18	0	0.55	0.00
BCC	83	82.08	1.01	7	7.62	0.92	4	6.19	0.65
Breast	11	17.61	0.62	3	1.89	1.59	2	1.98	1.01
r	1	2.47	0.40	0	0.30	0.00	1	0.26	3.78
Uterus	2	2.76	0.72	0	0.38	0.00	0	0.52	0.00
Colon	2	7.11	0.28	0	0.84	0.00	0	0.86	0.00

SIR = Standard Incidence Ratio, BCC = Basal cell carcinoma, cuSCC = Cutaneous squamous cell carcinoma.

**Table 5:** Observed and expected cancers in the whole Swedish cohort in a forest plot (Series 2).

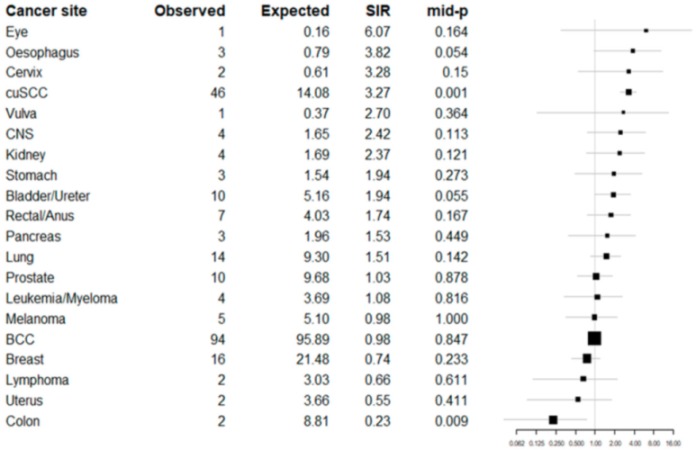

BCC = Basal cell carcinoma, cuSCC = Cutaneous squamous cell carcinoma.
